# In-play forecasting in football using event and positional data

**DOI:** 10.1038/s41598-021-03157-3

**Published:** 2021-12-17

**Authors:** Maximilian Klemp, Fabian Wunderlich, Daniel Memmert

**Affiliations:** Institute of Training and Computer Science in Sport, German Sport University Cologne, Am Sportpark Müngersdorf 6, 50933 Cologne, Germany

**Keywords:** Applied mathematics, Scientific data, Statistics

## Abstract

Two highly relevant aspects of football, namely forecasting of results and performance analysis by means of performance indicators, are combined in the present study by analysing the value of in-play information in terms of event and positional data in forecasting the further course of football matches. Event and positional data from 50 matches, including more than 300 million datapoints were used to extract a total of 18 performance indicators. Moreover, goals from more than 30,000 additional matches have been analysed. Results suggest that surprisingly goals do not possess any relevant informative value on the further course of a match, if controlling for pre-game market expectation by means of betting odds. Performance indicators based on event and positional data have been shown to possess more informative value than goals, but still are not sufficient to reveal significant predictive value in-play. The present results are relevant to match analysts and bookmakers who should not overestimate the value of in-play information when explaining match performance or compiling in-play betting odds. Moreover, the framework presented in the present study has methodological implications for performance analysis in football, as it suggests that researchers should increasingly segment matches by scoreline and control carefully for general team strength.

## Introduction

Forecasting of results^1,2^ and performance analysis using event and positional data^3,4^ are two highly relevant and highly topical strands of research with regard to data-driven analysis in the game of football. However, until now, event and positional data have surprisingly not been used in the context of in-play forecasting models in football. The present paper presents a framework for a joint evaluation of both aspects, as well as empirical evidence on the usability of in-play information for forecasting purposes.

Event data and positional data from football matches aim at capturing all events and movements on the pitch and are comprehensively studied in sports science^5,6^. Event data provides a detailed and ordered sequence of all the player’s actions during the match, such as passes, shots, or tackles^7^. Although efforts to automatically detect events from video^8^ or positional data^9^ are undertaken, the most reliable and most widely used approach remains to be manual annotation by expert video analysts, supported by human and computer-based quality control^10–12^. Each event is described by the time and location where the action took place on the field as well as the event type. Depending on the data provider, additional information such as a subtype or the outcome of the event is given. By aggregating event data to count-based metrics, the technical performance of players or teams in a match can be assessed and related to indicators of success. Recent contributions followed this approach to establish player evaluation frameworks^10,11,13,14^. In match analysis research, metrics derived from event data have been utilized to explain teams’ success in a match^3,15^ throughout a season^16–18^, and to examine playing styles^19^.

Positional data, sometimes also referred to as tracking data, reflects information on the x/y-coordinates of all players and the ball at each observed point in time (usually 25 frames per second). In football, positional data in training is mostly gathered by Global Positioning Systems or Local Positioning Systems. In contrast, match positional data is tracked by recording video data with multiple cameras from several positions and applying computer vision algorithms and triangulation to get the players’ and ball’s positions^20^. Positional data has been used to examine players’ activity profiles with respect to playing positions^21^ or success over a season^22^, reporting the distance covered or efforts undertaken. Besides these physical parameters, tactical analyses by means of positional data have scrutinized the teams’ positioning on the field and, following from that, tried to quantify the space controlled by players and teams^23^. Here, a particular area is considered under the control of a player if that player can reach any point in that area before anyone else. These efforts resulted in the proposal of the pitch control parameter^24^, which has since been the subject of further research^25^. While event data is relatively widely available and notable amounts of data have been published^10,11^, tracking data is less available and, therefore, recent research using tracking data has mostly considered only small sample sizes^14^.

Another aspect of event and positional data, that is relevant to bookmakers and match analysts, but has not been tackled so far, is the value of such data in forecasting further success. The literature on forecasting in football (for an overview, see^1,2^ is driven by the idea of developing and testing models with the intention to estimate the outcomes of matches in advance. Mathematically, this includes efforts to find models that accurately reflect the inherent processes in football matches, such as Poisson models, (Koopman, et al.^26^; Maher^27^), regression models^28,29^, birth process models^30^ or, more recently, increasingly machine learning methods^31^.

The interest in research on football forecasting, however, is also driven by economic considerations, such as understanding the mechanisms of the sports betting market^32–34^ or financially profiting from it by identifying profitable betting strategies^26,35^. The sports betting market “has been the subject of considerable structural change caused by the growth of Internet betting alternatives to traditional bookmakers”^33^, which, according to the authors, implied an increasing competitive pressure resulting in more accurate bookmaker forecasts. A more recent significant change in the betting market is the increased importance of in-play betting, which refers to bets placed during a football match in progress. However, the forecasting literature does not seem to have kept pace with this development, as the question of effective in-play forecasting in football has not been sufficiently addressed. To the best of our knowledge, the work of Zou et al.^36^ as well as the work of Robberechts et al.^37^ are the only articles focussing precisely on this topic so far. While both studies adopted a Bayesian approach to predict the further course of a match based on in-play information, they do not report, how valuable in-play information is for forecasting the outcome of the match, compared to a baseline of pre-game expectation.

The deduction of in-play forecasting models from existing pre-play models is relatively straight forward, as the existing models and given pre-game information can simply be transferred to an adjusted remaining match time. However, the crucial question of genuine in-play forecasting is whether information from the previous course of the match is valuable to improve upon forecasts based on pre-game information. Zou et al.^36^ claim promising results in this regard, while other statistical investigations of football matches suggest that in-play effects on goal scoring (i.e. deviations from constant scoring rates), if existent, can be considered very small^38,39^. While the previously mentioned analyses relied solely on the information of goals during matches, data-richer in-match information based on event and positional data has the potential to possess a higher value in in-play forecasting.

The present paper thus contributes to an improved understanding of the extent to which event and positional data can be valuable in in-play forecasting of football matches. Specifically, it shall be examined, whether previously used models can be meaningfully improved by introducing indicators extracted from these data as predictors. While event and position data have been shown to possess value in describing teams’ performance on the one hand and in-play information has been used to forecast the further course of a match on the other, to the best of our knowledge, this is the first paper to focus on combining both these strands of research in football analysis.

## Method

### Data

The data used for the present analysis consists of two separate datasets. This approach was chosen because the number of matches for which position and event data are available is limited, while more fundamental information is accessible for a much higher number of matches. In this way, it was possible to establish a reliable baseline of the predictive value of widely available indicators, before testing the added value of position and event data against this baseline. The first dataset includes matches from 10 seasons (07/08–16/17) in 10 of the strongest European football leagues (first divisions of England, Spain, Germany, Italy, France, Portugal, Belgium, Turkey, Netherlands and Greece). For each match, the number of goals scored by each team in each half as well as average betting odds for the outcomes home, draw, away, over 2.5 goals and under 2.5 goals are available. This results in a total dataset of 31,912 matches and is split into five seasons of in-sample data (15,844 matches) and five seasons of out-of-sample data (16,068 matches) for analysis. Data have been obtained from https://football-data.co.uk.

The second dataset consists of position data and event data from 50 matches from the German Bundesliga in the season 2014/2015. The matches used stemmed from 31 distinct matchdays in the season, and 11 different teams played in these matches. For these 50 matches, positional data had been gathered through a semiautomatic optical tracking system (VISTRACK, by Impire Corp., Germany) at a sampling rate of 25 Hz. The measurement error of this system in tracking players’ positions (expressed as root mean squared error) was shown to be less than one meter for different activities^40^. Event data was gathered by manual video tagging of matches, followed by automated and manual post-processing^41^. The inter-operator reliability of this method was shown to be very good for the number of team events detected (with *kappa* values of 0.86 to 0.94) and individual events (Intra-class correlation of 0.96 to 0.99)^41^. The final event dataset consisted of 77,671 events (on average 1,553 ± 95 events per match), while the position dataset in total spanned 7,004,231 rows and 46 columns (x- and y-coordinates of 22 players and the ball; on average 140,085 ± 2406 rows per match) which results in a total of more than 300 million data points. These numbers indicate the high volume and complexity of the data used, which on the one hand, result in a high amount of information, on the other hand, in considerable challenges in the aggregation to indicators on the match level. Following the volume and complexity of the data produced during a football match, handling these can be termed an endeavour in Big Data analysis.

The study was approved by the local ethics committee at the German Sport University Cologne (DSHS 093/2017) and fully complies with the guidelines stated in the Declaration of Helsinki.

### Performance indicators

#### Technical indicators

Several, count-based indicators of technical performance were extracted from event data per team per half. Following previous work^3,16,17,42^, the numbers of shots, passes, short passes, long passes, crosses, throw-ins and clearances were gathered.

#### Physical indicators

From positional data, different indicators of physical performance were calculated. For both teams and both halves, we collected total running distance^21^, running distance covered while the team is in possession of the ball and running distance covered while the team is not in possession of the ball^22^. Unlike previous papers, we normalized the distance covered in and out of possession by dividing it through the time spent in and out of possession for both teams, respectively, in order to avoid a confounding effect of overall ball possession rates. We also calculated high-speed running distance (distance covered at speeds higher than 14.4 $\frac{km}{h}$) based on commonly used velocity thresholds^21^.

#### Tactical indicators

Furthermore, indicators of tactical performance were extracted from positional data utilising pitch control models and ball metrics. We calculated the area of the pitch controlled by both teams during the two halves. The area controlled per player was computed following the methods described by Kim^24^ and, to account for different pitch sizes, calculated the relative space on the pitch controlled by both teams. We computed space control on the whole pitch as well as space control in the defensive third, midfield third and attacking third for both teams, respectively^43^. Additionally, ball possession rates per team were extracted^3^ and, for the first time, we also extracted the distance travelled by the ball during both teams’ possession (normalized by the teams’ time in possession).

An overview of all performance indicators and their abbreviations can be found in Table 1.

**Table 1:** Performance indicators used in this study with descriptions, abbreviations and example references.

Performance indicator [abbreviation]	Description	Example Reference
**Technical performance**		
Shots [SHOT]	Number of shots	^16^
Passes [PASS]	Number of passes	^16^
Short passes [SPASS]	Number of short passes	^3^
Long passes [LPASS]	Number of long passes	^3^
Crosses [CROSS]	Number of crosses	^3^
Throw-Ins [THROW]	Number of throw-ins	^42^
Clearances [CLEAR]	Number of clearances	^3^
Fouls [FOUL]	Number of fouls committed	^3^
**Physical performance**		
Running distance [RD]	Total distance covered by all players	^21^
In-possession running distance [RD_IP]	Total distance covered by players while team in possession	^22^
Out-of-possession running distance [RD_OOP]	Total distance covered by players while team not in possession	^22^
High-speed running distance [RD_HS]	Total distance covered by players faster than 14.4 km•h^−1^	^21^
**Tactical performance**		
Ball possession [BP]	Relative ball possession rate	^3^
Ball distance [BD]	Total distance covered by the ball while the respective team in possession	Analysed for the first time
Space control [SC]	Proportion of the pitch controlled by the respective teams by means of Voronoi diagrams	^43^
Space control attacking third [SC_ATT]	Proportion of the attacking third controlled by the respective teams by means of Voronoi diagrams	^43^
Space control midfield third [SC_MID]	Proportion of the midfield third controlled by the respective teams by means of Voronoi diagrams	^43^
Space control defensive third [SC_DEF]	Proportion of the defensive third controlled by the respective teams by means of Voronoi diagrams	^43^

### Statistical framework

#### General idea of in-play forecasting

The idea of in-play forecasting is to use information getting available over the course of a match to forecast the further course of this particular match. In the present study, the value of information becoming available during the first half to forecast the outcome of the second half of the match is tested. To design a statistical framework that honestly tests for in-play forecasting, some points need to be considered. First, the subject of forecasting is not the final outcome of a match, but only the isolated outcome of the second half. If the halftime score is 3–0, the home team naturally has a highly improved probability of winning the match, and the halftime result will help improve the forecast for the outcome drastically. However, this is just a direct consequence of the current scoreline and not what we consider to be in-play forecasting. If the information of the halftime score of 3–0 implies that the home team also has a high probability of winning the second half, this would be valuable information for in-play forecasting. Second, the model needs to control for pre-game expectation as even before the match, the isolated outcome of the second half can be modelled and forecasted. We only consider the first half to have actual value for in-play forecasting if the first half adds additional value to the current pre-game expectation for the second half.

#### Forecasting accuracy

The first dataset covering a large sample size is used to test the ability of pre-game information (betting odds) and the most basic in-play information (goals in the first half) to forecast the outcome of the second half in terms of home win, draw and away win. Framing the outcome of the match with respect to these categories is a common procedure in forecasting^29,31,35^ since the betting market is structured likewise and the home advantage is already taken into account in this way. Five different models are compared, including two simple benchmark models as well as three ordered logistic regression models. The model **UNI** uses a uniform forecast of 33.33% for the three possible outcomes for each match, while the model **FRQ** uses the observed frequency of the three outcomes in the in-sample data as the forecast for each match in the out-of-sample data. Moreover, three different ordered logistic regression models are fitted in-sample using the second half result as the dependent variable. The model **PROB** uses the probability of a home win and the probability of an away win as obtained from the betting odds as the independent variables (see Wunderlich and Memmert^44^ for the calculation used to obtain probabilities from betting odds). The model **GOAL** uses the goal difference at the end of the first half (i.e. the halftime result) as the independent variable, and the model **BOTH** uses both the probabilities and the goal difference. These three fitted ordered logistic regression models are then used to forecast the outcomes in the out-of-sample dataset. For more information on UNI and FRQ as benchmark models as well as on using ordered logistic regression for forecasting outcomes in football, we refer to Hvattum and Arntzen^29^.

In summary, the five models represent different levels and types of information, namely no information at all (UNI), only football-specific, but no match specific information (FRQ), only in-play information from the first half (GOAL), only pre-game information (PROB) or both pre-game and in-play information (BOTH). The forecasting accuracy of all five models is measured utilising the widely used rank probability score^45^, and the accuracy of various models is compared by performing a paired t-test for each pair of these models.

#### Correlation analysis

On the one hand, event and positional data contain an incredible wealth of data points per match, as has already been outlined before. On the other hand, the number of available matches with positional data is limited, in particular, if compared to the almost unlimited availability of pure match results in terms of goals. This small sample size is a challenge, which is even intensified by the fact that very different match outcomes in terms of goals (i.e. 5–1 and 1–0) are assigned to the same category of a home win, and as such, the outcome in terms of home win, draw and away win is even more prone to randomness. For this reason, the above method cannot simply be applied to the complete set of performance indicators. Moreover, we would like to get a better understanding of the interaction of the performance indicators with overall team strength and the course of the match, as well as the ability of these indicators to explain and forecast success. Therefore, a correlation analysis including four different correlation coefficients is performed for each performance indicator.

We denote ${p}_{H}$ and ${p}_{A}$ as the probability for the home and the away team to win the match as obtained from the betting odds. Based on the fact that betting odds have an excellent predictive value in football^29,31,33,46^, these probabilities can be utilised as a highly accurate measure of relative team strength^47^. The performance indicators are denoted as ${i}_{H}^{x}$ for the home team and ${i}_{A}^{x}$ for the away team, where $x=1$ represents the first half and $x=2$ represents the second half. Analogously, the number of goals scored by each team are denoted as ${g}_{H}^{x}$ and ${g}_{A}^{x}$.

As a minimum requirement to assume predictive power in a performance indicator, it should in some way be related to immediate success or expected success (i.e. team strength), which is tested by two measures.

##### Strength dependence

Strength dependence is defined as:

$$r ({p}_{H}-{p}_{A}, {i}_{H}^{1}-{i}_{A}^{1})$$

which means that the correlation between the strength difference and the difference in the respective performance indicator with respect to the first half is calculated. Thus, a high positive correlation coefficient means that there is a strong connection between the strength of a team (i.e. the expected success) and the respective performance indicator.

##### Explanatory power

Explanatory power is defined as:

$$r ({i}_{H}^{1}-{i}_{A}^{1}, {g}_{H}^{1}-{g}_{A}^{1})$$

which means that the correlation between the respective performance indicator and the number of goals, both with respect to the first half is calculated. By doing this, the relationship between a performance indicator and the success of a team in the same half (i.e. the immediate success) can be tested. This approach is a standard approach in performance analysis in football to measure the importance of performance indicators^48^, however, it can be highly confounded by the course of a match, which means that it remains unclear whether the performance indicator explains success or is just the consequence of the current scoreline and the teams' resulting tactical approaches.

In addition, two measures of predictive performance are introduced, as a relation to team strength or success does not necessarily imply predictive value. Therefore, the connection between performance measures in the first half and success in the second half is analysed.

##### Predictive power

Predictive power is defined as:

$$r ({i}_{H}^{1}-{i}_{A}^{1}, {g}_{H}^{2}-{g}_{A}^{2})$$

which is similar to the explanatory power, but uses the outcome of the second half, which has two advantages. First, it is possible that a positive performance in the first half is connected to success in the first half, but not to the performance and success in the second half. Second, when comparing across different halves, we circumvent the problem mentioned above that the outcome of the first half is highly associated with the scoreline throughout the first half.

##### Predictive overperformance

Predictive power already comes close to the idea of in-play forecasting; however, it still does not control for pre-game information on the team strengths. If we assume that stronger teams are more successful across the whole match and show a higher performance concerning a particular performance indicator across the entire match, this would imply a positive value for the predictive performance. In order to test for the real value of the first half information in terms of in-play forecasting, the approach needs to control for the pre-game expectation. This is done by calculating an expectation for goals and performance indicators based on the pre-game winning probabilities.

The expected values of the performance indicator for the first half are denoted as $E\_{i}_{H}^{1}$ for the home team and $E\_{i}_{A}^{1}$ for the away team and analogously for the expected goals $E\_{g}_{H}^{2}$ and $E\_{g}_{A}^{2}$ in the second half. To estimate these numbers, we use these four expectations as dependent variables in regression models and use the probability of a home win, the probability of an away win and the probability of over 2.5 goals as independent variables. Count variables (SHOT, PASS, SPASS, LPASS, CROSS, THROW, CLEAR, FOUL) are modelled by Poisson regressions, while other variables are modelled by linear regressions (RD, RD_IP, RD_OOP, RD_HS, BD). For those variables representing a percentage (BP, SC, SC_DEF, SC_MID, SC_ATT), only $E\_{i}_{H}^{1}$ needs to be calculated while the expectation of the away team is consequently ${E\_}_{{i}_{A}^{1}}=1-E\_{i}_{H}^{1}$. Please note that in slight variation to this formula, the expectation of SC_DEF for one team is calculated as the counterpart to the expectation of SC_ATT for the other team and vice versa. The regression for goals is not fitted based on the dataset of 50 matches but on the larger in-sample dataset of 15,844 matches.

The predictive overperformance is then defined as:

$$r \left({{(E\_}_{i}}_{H}^{1}-{{E\_}_{i}}_{A}^{1}\right)-\left({i}_{H}^{1}-{i}_{A}^{1}\right), \left({{E}_{g}}_{H}^{2}-{{E}_{g}}_{A}^{2}\right)-({g}_{H}^{2}-{g}_{A}^{2}))$$

which means that the overperformance with regard to the performance indicator in the first half is correlated to the overperformance in terms of goals in the second half. In case of a highly positive correlation, this measure is evidence that a high performance with regard to the performance indicator has predictive value for the second half, even if controlling for team strength.

## Results

### Descriptive statistics

Descriptive statistics for all performance indicators, including mean, standard deviation, minimum and maximum for home and away teams, are summarized in Table 2.

**Table 2:** Descriptive Statistics for all Performance Indicators.

Performance indicator	Home				Away			
	Mean	SD	Min	Max	Mean	SD	Min	Max
**Technical performance**								
SHOT [#]	7.3	3.5	2	16	5.3	2.5	0	11
PASS [#]	233.8	65.1	105	419	210.5	57.4	99	357
SPASS [#]	210.6	65.3	85	396	187.9	57.1	80	332
LPASS [#]	23.2	5	10	34	22.6	5.8	10	41
CROSS [#]	8.3	3.9	1	18	6.7	3.5	2	18
THROW [#]	13.1	4.5	5	27	12.2	3.6	5	20
CLEAR [#]	29.8	7	17	43	33	9.4	13	52
FOUL [#]	6.6	2.7	2	13	7.5	2.9	2	15
**Physical performance**								
RD [m]	57,772.7	2,397.5	51,199.7	63,578.8	57,407.6	2,559.4	51,166.6	64,215.2
RD_IP [m/min]	1,242.5	73.6	1,068.6	1,440.3	1,227	73.2	1,069.4	1,408
RD_OOP [m/min]	1,286.9	88.3	1,098.9	1,547.5	1,284.4	96.6	1,088.9	1,554.7
RD_HS [m]	13,627.4	1,355.8	10,713.1	16,909.8	13,522.1	1,335.7	11,100.8	16,466.5
**Tactical performance**								
BP [%]	50.9	5.7	38.1	62	49.1	5.7	38	61.9
BD [m/min]	163	16.1	127.5	203.7	157.7	14.3	132.3	190.7
SC [%]	51.7	4.5	41.6	62.8	48.3	4.5	37.2	58.4
SC_ATT [%]	16.3	4.1	9	25.2	13.3	3.7	4.9	23
SC_MID [%]	51.9	6.3	34.6	68.2	48.1	6.3	31.8	65.4
SC_DEF [%]	86.7	3.7	77	95.1	83.7	4.1	74.8	91

*SD* standard deviation, *Min* minimum, *Max* maximum, units are presented in brackets where # refers to count variables.

### Predictive power

#### Forecasting accuracy

Table 3 illustrates results for the accuracy of five models in forecasting the outcomes of the second half.

**Table 3:** Results for various models forecasting the outcome of the second half in terms of home win, draw or away win.

Model	RPS	UNI	FRQ	GOAL	PROB
UNI	**0.2207**	–	–	–	–
FRQ	**0.2171**	< 0.0001*	–	–	–
GOAL	**0.2161**	< 0.0001*	< 0.0001*	–	–
PROB	**0.1992**	< 0.0001*	< 0.0001*	< 0.0001*	–
BOTH	**0.1990**	< 0.0001*	< 0.0001*	< 0.0001*	0.2862

*Significant at a 5% level.

As expectable, UNI has the worst predictive accuracy and is significantly outperformed by FRQ showing the second-worst result. Both naïve benchmark models are significantly outperformed by the three logistic regression models. Surprisingly, based on the first half goals, GOAL only mildly improves the forecasting accuracy of the benchmark model FRQ and is massively outperformed by PROB based on betting odds. This means that the betting odds reflecting the pre-game expectation possess a far higher predictive value than the goals reflecting basic in-play information. The model BOTH even fails to significantly outperform PROB, which means that when controlling for pre-game information, the in-game information in terms of goals seems not valuable for in-play forecasting at all. This is evidence that either in-play forecasting is hardly possible in general or that goals are too noisy and random-affected to gain sufficient value. The performance indicators will help to answer whether in-play forecasting becomes possible through the use of more sophisticated measures.

#### Correlation analysis

To provide a basis for comparison for the analysis of performance indicators, the correlation analysis was also performed for the number of goals. The strength dependence is 0.34, which is evidence for the obvious fact that stronger teams score more goals. Simultaneously, it demonstrates the relatively high randomness in goals that prevents an even more explicit connection. The explanatory power of goals is 1.00 being a direct consequence of the definition. The predictive power is 0.10, while the predictive overperformance is − 0.03, which is in line with the above results. It suggests some weak predictive power, which, however, almost completely disappears if controlling for team strength.

**Figure 1:**
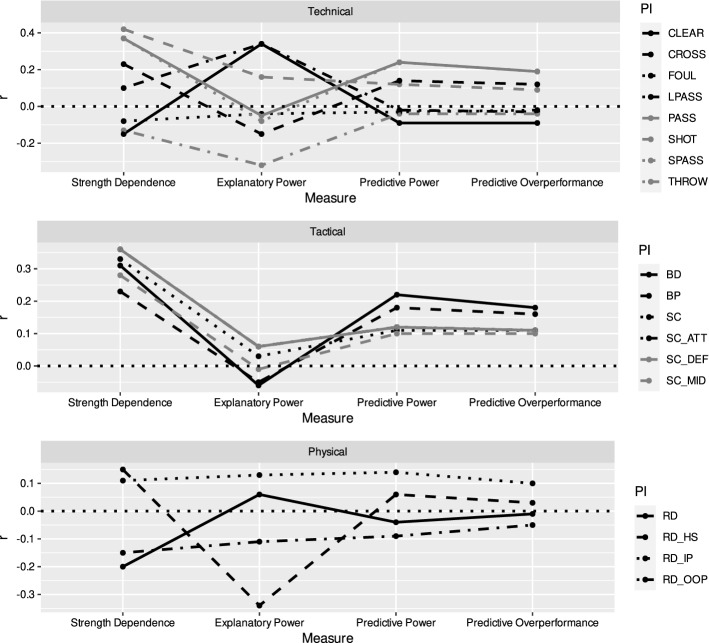
Correlation coefficients in the four measures of association for all performance indicators examined. Top panel shows technical performance indicators, middle panel shows tactical performance indicators and bottom panel shows physical performance indicators. Dashed horizontal line indicates a correlation coefficient of 0. Please refer to Method for a detailed description of the measures and performance indicators.

Table 4 reports the four correlation coefficients for each of the 18 performance indicators. Additionally, Fig. 1 illustrates the predictive value of the different performance indicators by showing how the correlation coefficients change when using the different measures of association examined.

**Table 4:** Correlations for various performance indicators.

Technical performance indicators								
	SHOT	PASS	SPASS	LPASS	CROSS	THROW	CLEAR	FOUL
Strength dependence	0.42**	0.37**	0.37**	0.10	**0.23**	− 0.13	− **0.15**	− 0.08
Explanatory power	**0.16**	− 0.05	− 0.08	0.34 **	− **0.15**	− 0.32**	0.34**	− 0.04
Predictive power	0.12	0.24*	0.24*	− 0.02	0.14	− 0.04	− 0.09	− 0.03
Predictive overperformance	0.09	**0.19**	**0.19**	− 0.03	0.12	− 0.04	− 0.09	− 0.02

Physical performance indicators								
	*RD*	*RD_IP*	*RD_OOP*	*RD_HS*				
Strength dependence	− **0.20**	0.11	− **0.15**	**0.15**				
Explanatory power	0.06	0.13	− 0.11	− 0.34**				
Predictive power	− 0.04	0.14	− 0.09	0.06				
Predictive overperformance	− 0.01	0.10	− 0.05	0.03				

Tactical performance indicators								
	*BP*	*BD*	*SC*	*SC_ATT*	*SC_MID*	*SC_DEF*		
Strength dependence	**0.23**	0.31**	0.33**	0.36**	0.28**	0.36**		
Explanatory power	− 0.05	− 0.06	0.03	0.06	− 0.01	0.06		
Predictive power	**0.18**	**0.22**	0.11	0.12	0.10	0.12		
Predictive overperformance	**0.16**	**0.18**	0.11	0.11	0.10	0.11		

**Significant at a 5% level, * significant at a 10% level, correlation coefficients with |r|> = 0.15 are highlighted in bold.

A variety of performance indicators are found to have a significant relationship to team strength. This refers to shots, passes, short passes as well as the ball distance and the four indicators of space control. The highest non-significant correlations are found for ball possession, crosses, clearances as well as running distance, out-of-possession running distance, and high-speed running distance. While most of the performance indicators are positively correlated, some exceptions show negative values. One example is running distance, which means that weaker teams generally need to run more than stronger teams. This is in line with previous research showing that more successful teams are covering less distance^49^. At first glance, it might come as a surprise that some performance indicators have an even higher correlation than the goals that—by definition—should be the clearest indicator of success. However, this can be easily explained by the fact that these performance indicators occur more frequently than goals^50,51^ and thus are less susceptible to randomness.

Results with regard to the explanatory power underline the problem of using this approach. Significant positive relationships are now found for long passes and clearances and significant negative relationships for throw-ins and high-speed running distance. For several performance indicators the direction or magnitude of effects seem to be in conflict with the strength dependence, which is very likely to be a reason of the confounding influence of the current scoreline. For example, clearances as an action taken in defence and under high pressure are rather performed by weak teams. At the same time, clearances are negatively correlated to the immediate success in that half, which can be assumed to be driven by more defensive tactics of teams currently leading.

With regard to the predictive power and predictive overperformance, promising results are found for passes, short passes, ball possession and ball distance. While passes and short passes are significant with regard to the predictive power at a 10% level, all variables fail to reach significance with regard to the predictive overperformance. However, if compared to goals, those performance indicators have higher correlations and seem to be more promising for in-play forecasting purposes.

## Discussion

The present study has presented a framework to distinguish more clearly between strength, performance and success in football. This framework is useful to analyse the predictive value of in-play information, but also revealed several insights related to performance analysis in football. The major result in this regard is the remarkable differences between strength dependence, explanatory power and predictive power for the majority of variables. Two different aspects can be considered responsible for this result and, at the same time, illustrate the highly misleading character of the common use of explanatory power in performance analysis.

First, performance indicators might rather reflect the general team strength than the specific performance in the respective match. This is corroborated by the finding that no performance indicator revealed a statistically significant relationship with regard to predictive overperformance (the correlation between a team’s performance in the first half and goals scored in the second half controlled for a priori expectation of team strength). At the same time, the team strength (known before the match) shows statistically significant correlations with many variables related to the teams’ performance. In conclusion, the result that the teams’ in-play performance is suitable for both explaining and predicting goal-scoring success, can be attributed in large part to the teams’ overall strength. This team strength, which is already known before the start of the match, affects both the teams’ performance and their success in scoring goals. Therefore, it might act as a strongly confounding variable on the relationship between performance and success.

Second, performance indicators that should serve as an explanation for the result might, in fact, rather be a consequence of the current result (i.e., the scoreline). The scoreline is known to have a substantial impact on technical^52,53^ and physical performance^54^ and consequently a correlation between performance and success within a half or a match can either be explained by performance affecting the success or by scoreline (i.e. past success) affecting performance. To solve this issue, performance analysis should attempt to break down a match into smaller segments to control for the effect of the scoreline on both performance and success.

In summary, both team strength and scoreline appear to be important contextual factors affecting independent and dependent variables within a match analysis framework. Accordingly, performance analysts in football should take care to carefully control for the effect of both variables. As a prerequisite for this, matches should ideally be segmented into sequences of respectively equal scorelines prior to statistical analyses.

With regard to forecasting, the present study has presented the theoretical idea of in-play forecasting including an empirical analysis based on a variety of performance indicators. Theoretically, we have argued that in-play forecasting models need to control carefully for pre-game expectation, in order to genuinely quantify the predictive value of in-play information. The presented approach makes use of pre-game betting odds, which are known to possess an exceptionally high predictive value^29,33,46^. The analysis of goals revealed that betting odds before the match are far more accurate in forecasting the second half of football matches than the outcome of the first half. More strikingly, goals in the first half did not add any significant value to a forecasting model for the second half, once controlling for pre-game betting odds. To ensure that the forecasting accuracy results are not affected by the choice of statistical methods, we checked their robustness against the accuracy measure and inference test choice. No relevant changes to the results and conclusions occurred when applying ignorance scores instead of rank probability scores^55^ or bootstrapping methods^56^ instead of t-tests. In summary, the results suggest that goals do not enable conclusions to be drawn about the further course of a match and as such, are not sufficient information for in-play forecasting.

To gain insights into the question of whether the process of football matches is inherently unsuitable for in-play forecasting or goals are just a too noisy source of information, several technical, physical and tactical performance indicators based on event and positional data were analysed. This idea is consistent with a line of research in performance analysis in football aiming to find more suitable performance indicators than goals^4,14^. Results revealed some promising performance indicators, in particular passes, short passes, ball possession and ball distance. Still none of these parameters revealed significant predictive overperformance, which is further evidence for the difficulty of in-play forecasting, but at the same time can be considered a consequence of the limited number of matches with event and positional data. While 50 matches are already a large set of data with regard to positional data literature, including analyses with less than ten^57,58^ or even only one match^59^, it is by no means comparable to the large datasets of more than ten thousand matches evaluable in football forecasting related to goals^1,29,46^. For this reason, the present study will not be able to give conclusive answers neither to in-play forecasting nor to the use of event and positional data in forecasting. However, to the best of our knowledge, the analysis of such detailed performance indicators in football forecasting has never been done before. Further studies with a higher number of matches will be a fruitful domain of research to gain more insights into the value of performance indicators. Moreover, larger datasets will make it reasonably possible to use machine learning methods, for example, to combine all the different performance indicators into a single one. We would also encourage researchers to tackle whether such performance indicators are useful in traditional (i.e., non-in-play) forecasting tasks using indicators from previous matches to forecast future matches.

Finally, we acknowledge that our representation of event data ignores the sequential nature of match events. It should be noted that match events can not only be viewed as separate, discrete events but also as sequences of different actions, where each of them is affected by the preceding one and affecting the following one, thereby forming a continuous process. This representation could be the base for the examination of further indicators describing teams’ tactical performance and their relationship with match outcome.

## Conclusion

The present study has focused on the use of in-play information to forecast the further course of a football match. We presented evidence that pre-game information is far more valuable in forecasting football matches then in-play information in terms of goals. While performance indicators based on event and positional data have been shown to possess more informative value than goals, even these indicators were not sufficient to reveal significant predictive value in-play. This is surprising and valuable news to match analysts who should not overestimate the value of in-play information in explaining match performance and bookmakers who should not overestimate the effect of in-play information on the accurate calculation of in-play betting odds. In defining strength dependence, explanatory power, predictive power and predictive overperformance, we presented a valuable framework for in-play forecasting and performance analysis in football. We would like to encourage researchers to adopt our framework for analyses with larger sample sizes. This will not only allow more robust conclusions about the relationships between variables but could also enable the use of more sophisticated machine learning methods for in-play forecasting^31^ as well as for the computation of in-depth performance indicators to quantify player or team performance^10,11,14^. Moreover, as a consequence of this study, we would strongly encourage the idea of segmenting matches by scoreline when using event or positional data for forecasting or performance analysis in football.

## References

[1] Koopman SJ, Lit R (2019). Forecasting football match results in national league competitions using score-driven time series models. Int. J. Forecast..

[2] Wunderlich F, Memmert D (2020). Forecasting the outcomes of sports events: A review. Eur. J. Sport Sci..

[3] Lepschy H, Wäsche H, Woll A (2020). Success factors in football: An analysis of the German Bundesliga. Int. J. Perform. Anal. Sport.

[4] Rein R, Memmert D (2016). Big data and tactical analysis in elite soccer: Future challenges and opportunities for sports science. Springerplus.

[5] Memmert D (2018). Data analytics in football: Positional data collection, modelling and analysis.

[6] Memmert D, Lemmink K, Sampaio J (2017). Current Approaches to Tactical Performance Analyses in Soccer Using Position Data. Sports Medicine.

[7] Garnica-Caparrós M, Memmert D (2021). Understanding gender differences in professional European football through machine learning interpretability and match actions data. Sci. Rep..

[8] Ekin A, Tekalp AM, Mehrotra R (2003). Automatic soccer video analysis and summarization. IEEE Trans. Image Process..

[9] Tovinkere, V., & Qian, R.J. (2001). Detecting semantic events in soccer games: towards a complete solution. In *IEEE International Conference on Multimedia and Expo, 2001. ICME 2001* (pp. 833–836). IEEE. 10.1109/ICME.2001.1237851

[10] Pappalardo L, Cintia P, Ferragina P, Massucco E, Pedreschi D, Giannotti F (2019). PlayeRank. ACM Trans. Intell. Syste. Technol..

[11] Pappalardo L, Cintia P, Rossi A, Massucco E, Ferragina P, Pedreschi D, Giannotti F (2019). A public data set of spatio-temporal match events in soccer competitions. Sci. Data.

[12] Memmert D (2021). Match Analysis.

[13] Brooks, J., Kerr, M., & Guttag, J. (2016). Developing a Data-Driven Player Ranking in Soccer Using Predictive Model Weights. In B. Krishnapuram, M. Shah, A. Smola, C. Aggarwal, D. Shen, & R. Rastogi (Eds.), *Proceedings of the 22nd ACM SIGKDD International Conference on Knowledge Discovery and Data Mining* (pp. 49–55). New York, NY, USA: ACM. 10.1145/2939672.2939695

[14] Decroos, T., Bransen, L., van Haaren, J., & Davis, J. (2019). Actions Speak Louder than Goals. In A. Teredesai, V. Kumar, Y. Li, R. Rosales, E. Terzi, & G. Karypis (Eds.), *Proceedings of the 25th ACM SIGKDD International Conference on Knowledge Discovery & Data Mining* (pp. 1851–1861). New York, NY, USA: ACM. 10.1145/3292500.3330758

[15] Broich H, Mester J, Seifriz F, Yue Z (2014). Statistical analysis for the First Bundesliga in the current soccer season. Progr. Appl. Math..

[16] Brito Souza D, López-Del Campo R, Blanco-Pita H, Resta R, Del Coso J (2019). A new paradigm to understand success in professional football: analysis of match statistics in LaLiga for 8 complete seasons. Int. J. Perform. Anal. Sport.

[17] Lago-Ballesteros J, Lago-Peñas C (2010). Performance in team sports: Identifying the keys to success in Soccer. J. Hum. Kinet..

[18] Schauberger G, Groll A, Tutz G (2018). Analysis of the importance of on-field covariates in the German Bundesliga. J. Appl. Stat..

[19] Hewitt A, Greenham G, Norton K (2016). Game style in soccer: What is it and can we quantify it?. Int. J. Perform. Anal. Sport.

[20] Thomas G, Gade R, Moeslund TB, Carr P, Hilton A (2017). Computer vision for sports: Current applications and research topics. Comput. Vis. Image Underst..

[21] Bradley PS, Sheldon W, Wooster B, Olsen P, Boanas P, Krustrup P (2009). High-intensity running in English FA Premier League soccer matches. J. Sports Sci..

[22] Hoppe MW, Slomka M, Baumgart C, Weber H, Freiwald J (2015). Match running performance and success across a season in German Bundesliga soccer teams. Int. J. Sports Med..

[23] Taki, T., & Hasegawa, J. (2000, June). Visualization of dominant region in team games and its application to teamwork analysis. In *Proceedings Computer Graphics International 2000* (pp. 227–235). IEEE Comput. Soc. 10.1109/CGI.2000.852338

[24] Kim S (2004). Voronoi analysis of a soccer game. Nonlinear Anal..

[25] Spearman, W., Basye, A., Dick, G., Hotovy, R., & Pop, P. (2017). Physics-based modeling of pass probabilities in soccer. In *Proceeding of the 11th MIT Sloan Sports Analytics Conference*.

[26] Koopman SJ, Lit R (2015). A dynamic bivariate Poisson model for analysing and forecasting match results in the English Premier League. J. R. Stat. Soc. A. Stat. Soc..

[27] Maher MJ (1982). Modelling association football scores. Stat. Neerl..

[28] Goddard J, Asimakopoulos I (2004). Forecasting football results and the efficiency of fixed-odds betting. J. Forecast..

[29] Hvattum LM, Arntzen H (2010). Using ELO ratings for match result prediction in association football. Int. J. Forecast..

[30] Dixon M, Robinson M (1998). A birth process model for association football matches. J. R. Stat. Soc. Ser. D (Stat.).

[31] Baboota R, Kaur H (2019). Predictive analysis and modelling football results using machine learning approach for English Premier League. Int. J. Forecast..

[32] Angelini G, de Angelis L (2019). Efficiency of online football betting markets. Int. J. Forecast..

[33] Forrest D, Goddard J, Simmons R (2005). Odds-setters as forecasters: The case of English football. Int. J. Forecast..

[34] Franck E, Verbeek E, Nüesch S (2010). Prediction accuracy of different market structures—bookmakers versus a betting exchange. Int. J. Forecast..

[35] Constantinou AC, Fenton NE, Neil M (2012). pi-football: A Bayesian network model for forecasting Association Football match outcomes. Knowl.-Based Syst..

[36] Zou Q, Song K, Shi J (2020). A Bayesian in-play prediction model for association football outcomes. Appl. Sci..

[37] Robberechts, P., Van Haaren, J., & Davis, J. (2021, August). A Bayesian Approach to In-Game Win Probability in Soccer. *Proceedings of the 27th ACM SIGKDD Conference on Knowledge Discovery & Data Mining* (pp. 3512–3521).

[38] Heuer A, Müller C, Rubner O (2010). Soccer: Is scoring goals a predictable Poissonian process?. EPL (Europhysics Letters).

[39] Heuer A, Rubner O (2012). How does the past of a soccer match influence its future? Concepts and statistical analysis. PLoS ONE.

[40] Siegle M, Stevens T, Lames M (2013). Design of an accuracy study for position detection in football. J. Sports Sci..

[41] Liu H, Hopkins W, Gómez AM, Molinuevo SJ (2013). Inter-operator reliability of live football match statistics from OPTA Sportsdata. Int. J. Perform. Anal. Sport.

[42] Taylor JB, Mellalieu SD, James N, Shearer DA (2008). The influence of match location, quality of opposition, and match status on technical performance in professional association football. J. Sports Sci..

[43] Lorenzo-Martínez M, Rein R, Garnica-Caparrós M, Memmert D, Rey E (2020). The effect of substitutions on team tactical behavior in professional soccer. Res. Q. Exerc. Sport.

[44] Wunderlich F, Memmert D (2018). The betting odds rating system: Using soccer forecasts to forecast soccer. PLoS ONE.

[45] Constantinou AC, Fenton NE (2012). Solving the problem of inadequate scoring rules for assessing probabilistic football forecast models. J. Quant. Anal. Sports.

[46] Štrumbelj E, Šikonja MR (2010). Online bookmakers’ odds as forecasts: The case of European soccer leagues. Int. J. Forecast..

[47] Wunderlich F, Berge F, Memmert D, Rein R (2020). Almost a lottery: The influence of team strength on success in penalty shootouts. Int. J. Perform. Anal. Sport.

[48] Sarmento H, Marcelino R, Anguera MT, CampaniÇo J, Matos N, LeitÃo JC (2014). Match analysis in football: A systematic review. J. Sports Sci..

[49] Di Salvo V, Gregson W, Atkinson G, Tordoff P, Drust B (2009). Analysis of high intensity activity in Premier League soccer. Int. J. Sports Med..

[50] Pollard R, Reep C (1997). Measuring the effectiveness of playing strategies at soccer. J. R. Stat. Soc. Ser. D (the Statistician).

[51] Rein R, Raabe D, Memmert D (2017). "Which pass is better?" Novel approaches to assess passing effectiveness in elite soccer. Hum. Mov. Sci..

[52] Bradley PS, Lago-Peñas C, Rey E, Sampaio J (2014). The influence of situational variables on ball possession in the English Premier League. J. Sports Sci..

[53] Lago C, Martín R (2007). Determinants of possession of the ball in soccer. J. Sports Sci..

[54] Odonoghue P, Robinson G (2016). Score-line effect on work-rate in English FA Premier League soccer. Int. J. Perform. Anal. Sport.

[55] Wheatcroft, E. (2019, August 23). *Evaluating probabilistic forecasts of football matches: The case against the Ranked Probability Score*. Retrieved from http://arxiv.org/pdf/1908.08980v1

[56] Efron B, Tibshirani RJ (1994). An Introduction to the Bootstrap.

[57] Folgado H, Duarte R, Marques P, Sampaio J (2015). The effects of congested fixtures period on tactical and physical performance in elite football. J. Sports Sci..

[58] Tenga A, Zubillaga A, Caro O, Fradua L (2015). Explorative study on patterns of game structure in male and female matches from elite Spanish soccer. Int. J. Perform. Anal. Sport.

[59] Fernandez, J., & Bornn, L. (2018). Wide Open Spaces: A statistical technique for measuring space creation in professional soccer. In *Sloan Sports Analytics Conference*.

